# A Longitudinal Examination of the Hopelessness Theory of Depression in People Who Have Multiple Sclerosis

**DOI:** 10.1155/2015/190405

**Published:** 2015-07-28

**Authors:** I. I. Kneebone, S. Guerrier, E. Dunmore, E. Jones, C. Fife-Schaw

**Affiliations:** ^1^Graduate School of Health, University of Technology, Sydney, NSW 2007, Australia; ^2^Virgin Care NHS, Haslemere, Surrey GU27 2BJ, UK; ^3^Department of Social Psychology, London School of Economics, London WC2A 2AE, UK; ^4^Harrogate Grammar School, Harrogate, North Yorkshire HG2 0DZ, UK; ^5^School of Psychology, University of Surrey, Guildford, Surrey GU2 7XH, UK

## Abstract

*Purpose*. Hopelessness theory predicts that negative attributional style will interact with negative life events over time to predict depression. The intention of this study was to test this in a population who are at greater risk of negative life events, people with Multiple Sclerosis (MS). *Method*. Data, including measures of attributional style, negative life events, and depressive symptoms, were collected via postal survey in 3 phases, each one a year apart. *Results*. Responses were received from over 380 participants at each study phase. Negative attributional style was consistently able to predict future depressive symptoms at low to moderate levels of association; however, this ability was not sustained when depressive symptoms at Phase 1 were controlled for. No substantial evidence to support the hypothesised interaction of negative attributional style and negative life events was found. *Conclusions*. Findings were not supportive of the causal interaction proposed by the hopelessness theory of depression. Further work considering other time frames, using methods to prime attributional style before assessment and specifically assessing the hopelessness subtype of depression, may prove to be more fruitful. Intervention directly to address attributional style should also be considered.

## 1. Introduction

Multiple Sclerosis (MS) is an autoimmune disease that affects the central nervous system. Patients experience myelin and axonal destruction in the brain and spinal cord which often leads to substantial disability [1]. People who have MS can face numerous challenges including reduced social and vocational activity [2]. Such losses may well contribute to the high rates of depression identified in this population [3] and suggest that psychological models are appropriate to the understanding and indeed the treatment of their affective disorders.

The reformulated theory of learned helplessness [4] proposes that depression is a result of the attributions people make regarding events which affect them, specifically that an internal, stable, and global attributional style for negative events makes individuals vulnerable to the development of depression. Persons who view a negative event as caused by something to do with themselves (internal), as opposed to something unconnected to themselves (external), that is, due to something that affects a broad range of situations (global), rather than being confined to a narrow range of circumstances (specific) and due to factors which are long lived or recurrent (stable), rather than those that are short lived or intermittent (unstable), are considered more likely to become depressed. A central precept of this theory is that depression can derive from exposure to negative (stressful) life events* and *how these are considered by the individuals affected. People with MS are more likely than most to experience negative events as the disease can have an impact on independence, social, vocational, and family function. On this account, the reformulated theory of learned helplessness would appear particularly applicable to people with this condition. Two studies [5, 6] have considered the application of this theory of depression [4] to people with MS, demonstrating that stress interacts with attributional style to explain significant depressive symptoms. Kneebone and Dunmore [5] found global attributional style which interacted with negative events including life stressors and the time since last symptom exacerbation, to explain significant amounts of depression. More recently, another study found that stress, operationalised as daily hassles, mediated the relationship between attributional style and depression [6]. Unfortunately, a major limitation with these studies is that the support for the relationship has only been based on cross-sectional data. Hopelessness theory proposes a dynamic process in which attributional style interacts with negative events over time to predict depressive symptoms [4]. Cross-sectional findings could be explained by the attributional style expressed concurrently with reports of depressive symptoms, simply being indicative of current depressive state rather than being a salient style that is causal in the development of depression over time. Indeed, studies testing hopelessness theory in other populations have found it much harder to support when examined with longitudinal designs. For instance, while Golin et al. [7] were able to find evidence that negative attributional style predicted later depressive symptoms for college students, others have not [8–10]. The interaction between negative events and attributional style predicted by the model has also been found in such student samples [9–12] but not with psychiatric populations [13]. While work conducted with people who have medical problems has also found the predicted relationship between attributional style and depressive symptoms [14–16], the proposed interaction effect, or this effect emerging over time, was not present when tested for in these samples.

The current investigation is a longitudinal extension of a cross-sectional study [5] and thus reports on a test of hopelessness theory in people with MS which is able to take into consideration the temporal dimension and interaction prediction of the model by considering the influence of the relevant variables prospectively. It was expected that, consistent with hopelessness theory, attributional style for negative events would predict depressive symptoms and that negative attributional style would interact with negative life events to predict depressive symptoms prospectively, even when earlier symptoms were controlled for.

## 2. Method

Participants were recruited via an MS magazine published for residents of Great Britain and Northern Ireland. A brief article contained contact phone numbers for the study. Self-report data was collected by post in three phases at yearly intervals. All measures were administered at each phase.

### 2.1. Measures

#### 2.1.1. Center for Epidemiologic Studies Depression Scale (CES-D) [17]

The CES-D is a self-administered measure that requires those completing it to report on symptoms of depression experienced over the past week. Twenty symptoms are rated on a scale from “0” to “3.” Higher ratings indicate greater symptom frequency. Depression scores obtained from the scale can range from 0 to 60. The CES-D is relatively unaffected by somatic or illness variables [18] that have led to the questions regarding the validity of using other depression assessment instruments with people who have MS [19]. The CES-D has also been used in other studies considering depression in persons who have MS [20].

#### 2.1.2. Attributional Style Questionnaire-Survey (ASQ-S) [21]

The ASQ-S was developed to allow unsupervised assessment of attributional style. The questionnaire presents participants with 12 descriptions of negative events (e.g., “a friend is very angry with you,” “you have problems sleeping”). A principal cause for each negative event is then requested and rated on a seven-point scale (−3 to +3) for “stability” (“How likely is it the cause will continue to affect you?”) and “globality” (“Is the cause something that just affects…* negative event*…, or does it affect other areas of your life?”). Ratings are transformed to a “1” to “7” scale for data analyses. Stability of attributional style for negative events (STAB) scores is obtained by summation of all stability ratings and division by 12. The same procedure is followed to obtain globality of attributional style for negative events (GLOB) scores. This approach provides scores ranging from “1” to “7” for each attributional dimension. The scale has a simpler, clearer format than earlier questionnaires which lends it to use in survey research. In part, this has been achieved by removal of the less coherent attributional dimension (internality/externality), used in other measures.

#### 2.1.3. Time Since MS Exacerbation (TSE)

The amount of time since participants' last significant MS activity was one of two measures used to evaluate participants' experience of negative events. Participants were asked to indicate how long since they had their “last symptom flare up.” Several choices were offered from a number of alternatives (range from “1 week” to “over 12 months”).

#### 2.1.4. Recent Life Changes Questionnaire (RLCQ) [22]

The RLCQ considers more general stressors. It lists 74 significant events (e.g., “In-law problems,” “decreased income”). Each event is weighted for degree of stress devised on the basis proportionate scaling. Reliability of rankings based on divisions into age, gender, marital status, and education categories is between .84 and .96 (Spearman's *ρ*) [22].

#### 2.1.5. Disability

Disability was assessed using the* Functional Assessment Screening Questionnaire-Revised (FASQ-R)* [23, 24]. The FASQ-R was designed for use with medical patients having moderate disability. It assesses function with respect to 5 domains: personal care (e.g., “cutting toenails”), occupational (e.g., “concentrating for 15 minutes”), leisure (e.g., “playing your favourite sports”), transport (e.g., “boarding and exiting a bus”), and instrumental (e.g., “handling personal finances”). Use of the FASQ-R allowed the contribution of disability to depressive symptoms to be taken into account in the study.

## 3. Results

### 3.1. Participants

At Phase 1, 401 women and 94 men (*N* = 495), 65 years and under, were recruited. At Phase 2, 396 of these responded and at Phase 3, 386 responded, a retention rate of 80% and 78%, respectively. At initial recruitment, the age range was 22–65 years, the mean was 45.8 years, and standard deviation was 9.25. Forty-five percent reported that their MS was of the relapsing remitting type and 32.5% reported that their MS was of chronic progressive type. Eighteen percent were unable to report their MS type diagnosis. Well over half of the participants scored over 16 on the CES-D, the suggested cut-off for significant depressive symptoms on this instrument (M = 22.1, SD = 12.56, and range 0–59).

### 3.2. Association of Independent Variables with Depression

Using Pearson's *r* significant positive associations with depressive symptoms were found for GLOB, STAB, and RLCQ scores at Phase 1 and significant negative associations with TSE and FASQ-R [5].  Table 1 indicates that Phases 2 and 3 associations were consistent with this even when adjustment for multiple comparisons was made (*p* < .001).

### 3.3. Prediction of Depressive Symptoms from Attributional Style

As can be seen in Table 2, attributional style (GLOB and STAB) at Phase 1 was associated with depressive symptoms at Phases 2 and 3 and attributional style at Phase 2 was associated with depressive symptoms at Phase 3 (Pearson's *r*, adjusted *p* < .001). Higher levels of negative attributional style were associated with higher depressive symptoms up to 2 years later. Notably, however, these findings were not sustained when these data were examined using regression taking into account depression at Phase 1.

### 3.4. The Interaction of Negative Events and Attributional Style

According to the hopelessness theory of depression, the interaction between experiencing a negative life event and an individual's attributional style is more important in explaining variance in depressive symptoms than the experience of negative life events or having a negative attributional style on their own.

The interaction between negative life events (TSE and RCLQ) and negative attributional style was modelled using a linear mixed model with subjects as a random effect and attributional styles as fixed variables. The measure of current disability (FASQ-R) was entered into the regression as a covariate to control for its potential influence on depression scores. All measures and interaction terms were entered into the regression simultaneously. All variables were centred before inclusion in the model.

Two linear mixed models with interaction terms were performed. The first attempted to predict depressive symptoms from current disability (FASQ-R), most recent time since exacerbation (TSE) and the interactions between TSE and the two attributional variables from Phase 1, STAB 01 and GLOB 01. The second regression utilised the same variables but substituted the general stressors measure RLCQ for TSE.

In the first model, the interaction between TSE and STAB 01 was not significant though both negative attributional styles were predictive of depression scores as were time since exacerbation and disability level (see Table 3). For the second model, the interaction between recent life changes (RLCQ) and negative global attributions (GLOB 01) was significant though the effect size was very small (see Table 4). The main effects were similar to those found in the previous model.

## 4. Discussion

While negative attributional style was consistently associated with concurrent and future depressive symptoms (at low to moderate levels of association), this effect disappeared when Phase 1 depressive symptoms were controlled for. Highly limited evidence to support the interaction of negative attributional style and negative events to do this was found in this study. Only one of four expected interactions was evident and, given the marginal size of this interaction, confidence in this finding has to be low.

There may be several reasons, either independently or in concert, that explain the inability in the current study to demonstrate the hypothesised interactions. Firstly the interactions proposed may be insufficiently robust to be demonstrated. One reason the effects may be weak could be the fact that a large number of people with MS may have depressive symptoms as a direct result of their physical disease. The contribution of neuropathology to depression in this population is thought to be considerable, though a clear theoretical model to explain it is awaited [25]. A second reason the hypothesised interactions were not identified may lie in the way attributional style and depressive symptoms were measured in this study. Possibly a year is not the best time period over which to consider negative events interacting with attributional style to lead to depressive symptoms. There is also some support for the view that attributional styles need to be primed in certain ways to be adequately assessed [26, 27]. A standard measure of depressive symptoms was also used in the current study, consistent with our interest in depressive disorders. Unfortunately, this does not take into account the contention that the hopelessness theory of depression explains a particular subtype of depression and not all depression [28]. A general measure such as the CES-D is unlikely to be wholly representative of this subtype. Following on from this while the sample size was considerable, the self-recruitment procedure may also have meant some people with MS and depression may have been less likely to enroll, such as those with apathy/hopelessness to which attributional style may be particularly pertinent. Indeed, the general population of depressed patients with MS could have been symptomatologically biased and underestimated on account of this methodology. A population based study might overcome this concern. MS diagnosis was also based on self-report in this study. Future work might be conducted with persons diagnosed by a qualified neurologist using internationally accepted criteria. The propositions investigated here might differentially apply to different MS subtypes. We also did not consider the effects of disease altering pharmacological treatment, a variable of potential importance to depression in people with MS [29]. Finally, a more specific measure of MS related stress might have improved the study results.

While our findings are not supportive of strong causal interaction consistent with hopelessness theory, further investigations, considering other time frames, assessing attributional style once primed, using measures specific to hopelessness subtype of depression and with greater attention to neuropathological factors, may prove more promising. Further, the strongest test of the theory might be performed by direct manipulation of attributional style with monitoring to establish whether or not this affects depression levels in the face of ongoing negative life events; indeed, preliminary casework partially supports such an approach [30]. Importantly, this might circumvent the issue of autocorrelation for depression across studies, which made it difficult to detect effects using the current methodology.

## Figures and Tables

**Table 1:** Correlational Matrix for Independent Variables at Phases 2 and 3 with depressive symptoms (CES-D) at Phases 2 and 3.

	Variable	*N*	1	2	3	4	5	6
1	CES-D	394 (368)	—	.44^*∗*^ (.36^*∗*^)	.46^*∗*^ (.40^*∗*^)	.28^*∗*^ (.19^*∗*^)	−.14 (−.19^*∗*^)	−.24^*∗*^ (−.26^*∗*^)
2	STAB	226 (224)		—	.73^*∗*^ (.67^*∗*^)	.01 (−.02)	−.05 (−.04)	−.21 (−.19)
3	GLOB	225 (220)			—	.08 (.05)	−.06 (−.05)	−.21 (−.11)
4	RLCQ	298 (349)				—	−.20^*∗*^ (−.23^*∗*^)	.08 (.01)
5	TSE	396 (387)					—	.02 (−.01)
6	FASQ-R	324 (346)						—

*Note*. Figures in brackets represent values at Phase 3.

CES-D = Centre for Epidemiological Studies of Depression Scale, STAB = Stability of Attributions for Negative Events, GLOB = Globality of Attributions for Negative Events, RLCQ = Recent Life Change Questionnaire, TSE = Time Since Exacerbation, and FASQ-R = Functional Assessment Screening Questionnaire-Revised.

Correlational *N*'s range = 223–394 (variables at Phase 2) and 213–368 (variables at Phase 3).

^*∗*^ *p* > .001.

**Table 2:** Longitudinal Correlational Matrix for Independent Variables at Phases 1 and 2 with depressive symptoms at Phases 2 and 3.

	Variable		CES-D Phases 2 and 3					
			Independent variables Phases 1 and 2					
		*N*	1	2	3	4	5	6
1	CES-D 02	394	—					
2	CES-D 03	334	.66^*∗*^	—				
3	GLOB 01	230	.32^*∗*^	.35^*∗*^	—			
4	STAB 01	237	.30^*∗*^	.31^*∗*^	.67^*∗*^	—		
5	GLOB 02	223	.46^*∗*^	.33^*∗*^	.54^*∗*^	.42^*∗*^	—	
6	STAB 02	224	.44^*∗*^	.33^*∗*^	45^*∗*^	.56^*∗*^	.73^*∗*^	—

*Note*. CES-D 02 = Centre for Epidemiological Studies of Depression Scale, Phase 2, CES-D 03 = Centre for Epidemiological Studies of Depression Scale, Phase 3, GLOB 01 = Globality of Attributions for Negative Events, Phase 1, STAB 01 = Stability of Attributions for Negative Events, Phase 1, GLOB 02 = Globality of Attributions for Negative Events, Phase 2, and STAB 02 = Stability of Attributions for Negative Events, Phase 2.

Correlational *N* = 394 (CES-D Phase 2, independent variables Phase 1); correlational *N* = 334 (CES-D Phase 3, independent variables Phase 3); ^*∗*^ *p* < .001.

**Table 3:** Predicting Depressive Symptoms from the interaction between negative attributional styles and time since exacerbation of symptoms: summary of a linear mixed model.

	Variable	*B*	*t*	*p*	LLCI	ULCI
1	FASQ	−.24	−5.30	<.001	−.32	−.15
2	GLOB 01	.23	4.01	<.001	.12	.34
3	STAB 01	.14	2.42	.02	.03	.26
4	TSE	−.87	−4.01	<.001	−1.29	.44
5	TSE × GLOB 01	−.02	−.99	.32	−.07	.02
6	TSE × STAB 01	.02	.84	.41	−.03	.07

*Note*. Fixed effects coefficients are unstandardized. −2LL = 3708.694.

**Table 4:** Predicting Depressive Symptoms from the interaction between negative attributional styles and recent life changes: summary of a linear mixed model.

	Variable	*B*	*t*	*p*	LLCI	ULCI
1	FASQ	−.26	−5.66	<.001	−.35	−.17
2	GLOB 01	.20	3.55	<.001	.08	.30
3	STAB 01	.15	2.61	.009	.04	.26
4	RCLQ	.01	5.49	<.001	.01	.02
5	RCLQ × GLOB 01	.00^*∗*^	2.01	.05	.00	.00
6	RCLQ × STAB 01	−.00	−.71	.477	−.00	.00

*Note*. Fixed effects coefficients are unstandardized. −2LL = 3430.697.

^*∗*^To 2 dp, to 4 dp value = .0005.
