# Corrigendum to “A Longitudinal Examination of the Hopelessness Theory of Depression in People Who Have Multiple Sclerosis”

**DOI:** 10.1155/2020/1805958

**Published:** 2020-09-30

**Authors:** I. I. Kneebone, S. Guerrier, E. Dunmore, E. Jones, C. Fife-Schaw

**Affiliations:** ^1^Graduate School of Health, University of Technology, Sydney, NSW 2007, Australia; ^2^Virgin Care NHS, Haslemere, Surrey GU27 2BJ, UK; ^3^Department of Social Psychology, London School of Economics, London, WC2A 2AE, UK; ^4^Harrogate Grammar School, Harrogate, North Yorkshire HG2 0DZ, UK; ^5^School of Psychology, University of Surrey, Guildford, Surrey GU2 7XH, UK

Following the publication of the article titled “A Longitudinal Examination of the Hopelessness Theory of Depression in People Who Have Multiple Sclerosis” [1], the authors identified an error in the Center for Epidemiologic Studies Depression Scale (CESD) administered to participants. In item *10: “I felt* fearful” was printed as *“I felt tearful.”* The authors and handling editor are in agreement that the impact on the results and conclusions is negligible; however, this corrigendum serves to inform readers of this error.
